# Supplementary material for: Predicting visual function by interpreting a neuronal wiring diagram
Source: Nature. 2024 Oct 2;634(8032):113–23. doi: 10.1038/s41586-024-07953-5 (PMC11446822; doi:10.1038/s41586-024-07953-5)
Supplement: Supplementary file 6 — CRF and ERF predictions for individual TmY4 and TmY9 cells. Analogous to Supplementary Data 3, but for TmY target types. Shown are the top four monosynaptic pathways, the strongest pathway passing through each of the top ten intermediary types (ranking from Extended Data Fig. 7), and the trisynaptic pathway Tm1–TmY–Dm3–TmY (see the section entitled Prediction of spatial normalization). [file 41586_2024_7953_MOESM6_ESM.zip › DataS4/TmY9q/720575940628381962.pdf]

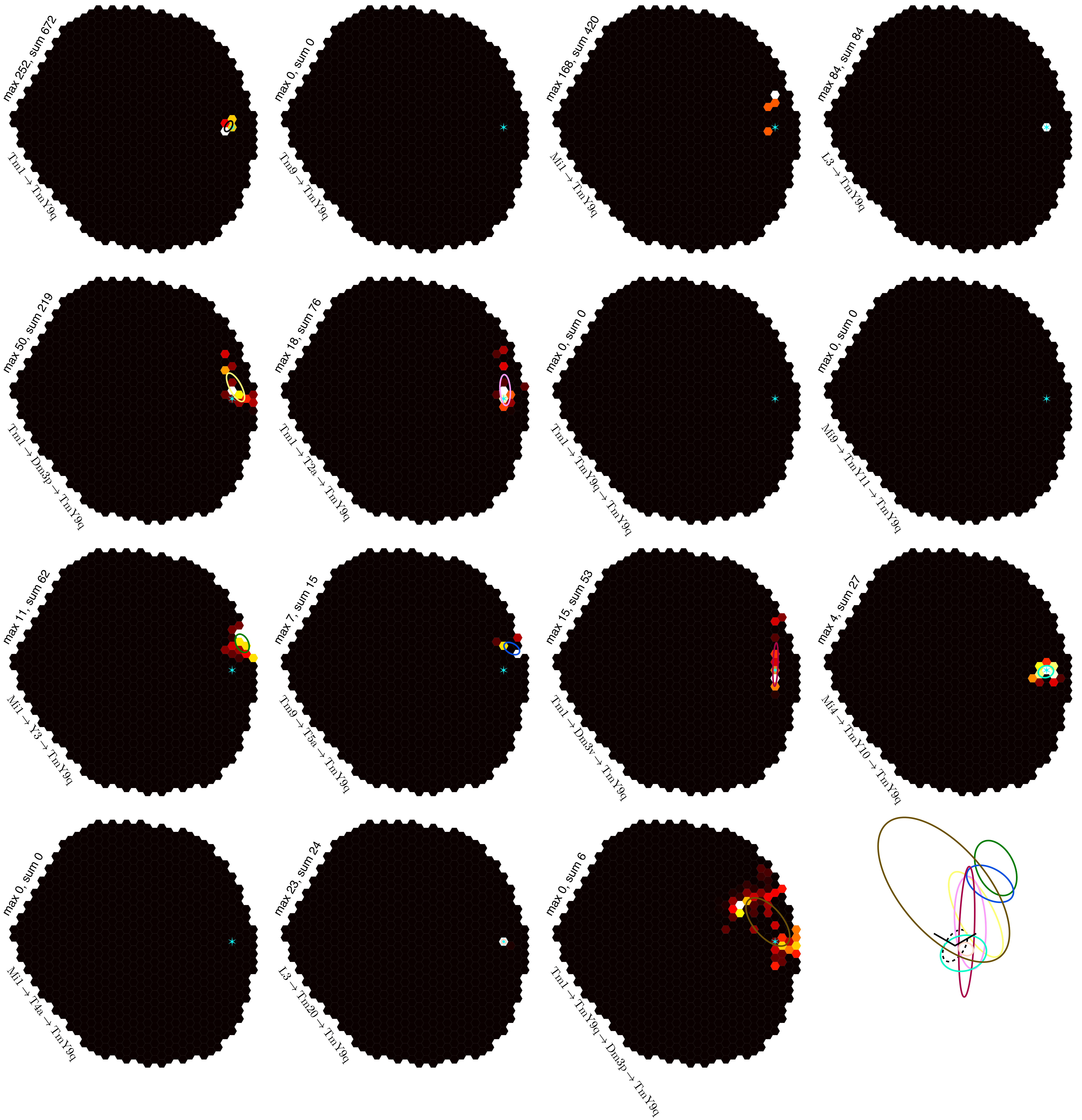
max 252, sum 672
Tm1 → TmY9q
max 0, sum 0
Tm9 → TmY9q
max 168, sum 420
Mi1 → TmY9q
max 84, sum 84
L3 → TmY9q
max 50, sum 219
Tm1 → Dm3p → TmY9q
max 18, sum 76
Tm1 → T2a → TmY9q
max 0, sum 0
Tm1 → TmY9q → TmY9q
max 0, sum 0
Mi9 → TmY11 → TmY9q
max 11, sum 62
Mi1 → Y3 → TmY9q
max 7, sum 15
Tm9 → T5a → TmY9q
max 15, sum 53
Tm1 → Dm3v → TmY9q
max 4, sum 27
Mi4 → TmY10 → TmY9q
max 0, sum 0
Mi1 → T4a → TmY9q
max 23, sum 24
L3 → Tm20 → TmY9q
max 0, sum 6
Tm1 → TmY9q → Dm3p → TmY9q
